# Eliminating female *Anopheles arabiensis* by spiking blood meals with toxicants as a sex separation method in the context of the sterile insect technique

**DOI:** 10.1186/1756-3305-6-197

**Published:** 2013-07-03

**Authors:** Hanano Yamada, Sharon M Soliban, Marc JB Vreysen, Dave D Chadee, Jeremie RL Gilles

**Affiliations:** 1 Insect Pest Control Laboratory, Joint FAO/IAEA Division of Nuclear Techniques in Food and Agriculture, International Atomic Energy Agency, Vienna, Austria; 2 Department of Life Sciences, University of the West Indies, St. Augustine, Trinidad

**Keywords:** *Anopheles arabiensis*, Ivermectin, Virbamec**®**, Female elimination, Male competitiveness, Sterilisation, Sterile insect technique

## Abstract

**Background:**

Ivermectin has longevity reducing effects in several insect species, including disease transmitting mosquitoes after feeding on hosts that have received ivermectin treatment. This has important implications in mosquito population control and thus the reduction of disease transmission. In addition, ivermectin could play an enormous role in mosquito control operations by its use in the female elimination process during mass-rearing, enabling the release of only sterile males in the context of the sterile insect technique (SIT).

**Methods:**

Blood meals were spiked with various toxicants and were then offered to adult *Anopheles arabiensis* and killing effects were observed. Varying concentrations of the most effective substance were then tested in subsequent trials to obtain an optimal dose for quick and total female elimination. The remaining males were mated with untreated virgin females to assess whether their mating efficiency had been compromised. The most promising substance at the optimal concentration was further tested on a larger number of adults, after they had been irradiated and partially sterilised as pupae with 70 Gy to evaluate the feasibility of the method in a mass-rearing, and SIT context. The males resulting from the latter trial were also checked for mating efficiency post treatments.

**Results:**

Ivermectin (Virbamec**®**) at a concentration of 7.5 ppm was chosen from the toxicants tested as sufficiently effective in eliminating all female *An. arabiensis* in 4 days, the shortest time required for female elimination of all chemicals tested. Mating efficiency of the non-blood feeding male mosquitoes was not compromised significantly compared to controls even when they were kept for a total of 4 days (from emergence) before theoretical release. The irradiation treatment did not affect overall female feeding behaviour in this setting, nor were the sterile males less competitive for mating with virgin females after the treatments than virgin sterile males that had not been in the ivermectin treatment environment.

**Conclusions:**

Spiking bloodmeals with ivermectin has shown potential as a viable treatment to eliminate female *An. arabiensis* from laboratory colonies although its practical use in a mass-rearing facility still needs to be tested.

## Background

Ivermectin is a macrocyclic lactone extract from the bacteria *Streptomyces avarnitalis* that acts at invertebrate glutamate-gated chloride channels [1] inhibiting neurotransmission and thereby affecting the nervous system and muscle function in parasites. This drug has proven to be effective for treating mammals against a broad range of nematodes and ectoparasites [2] and is used globally in animal husbandry practices. Ivermectin has been found safe for treatment of humans [3] and is currently being used with great success in mass drug administration (MDA) campaigns against onchocerciasis and lymphatic filariasis [4,5]. In addition, numerous reports have demonstrated the efficacy of ivermectin in significantly reducing the longevity of several arthropod species such as triatomine bugs, ticks, sandflies, and scabies [3].

In 1983 the effects of ivermectin on the tsetse fly was first documented by Distelmans *et al.*[6] who demonstrated high mortality in *Glossina tachinoides* flies that were fed on piglets treated with ivermectin. Other investigations have shown that the treatment of host animals with the active ingredient can achieve some level of tsetse population control [7], although the extent of this control was not further investigated in this study. Similar treatment effects were observed in mosquitoes following the MDA of ivermectin to humans: *An. gambiae* could be killed within 6 days after the administration of a standard dose of ivermectin (150 to 200 μg/kg body weight), indicating a mosquito population reducing effect that may have the potential to reduce malaria transmission [4]. However, this lethal effect was only apparent for one week, therefore this treatment modality alone will not serve as an effective intervention strategy [2,5].

Numerous studies have demonstrated the efficacy of ivermectin as a larvicide [8,9] and when fed to vertebrates have proven to be effective in reducing longevity to an array of mosquito species such as *Culex quinquefasciatus*[8-10], *An. gambiae*[5,11], *An. arabiensis*[11], *An. stephensi*[9,12], *Ae. aegypti*[9,10], *Ae. albopictus*[10], *An. sacharova*[12], *An. farauti*[13], and *An. quadrimaculatus*[14]. Reducing the longevity of medically important mosquitoes is a favourable side effect of medical treatments with ivermectin. In addition to this, it was hypothesized that ivermectin could be used for female elimination in mass-rearing facilities that produce male mosquitoes for use in incompatible insect technique (IIT) [15-18], and the sterile insect technique (SIT) [19] where only-male releases are permitted.

The SIT is an environmental friendly vector control tactic that becomes more effective under inversely density dependent conditions and is usually integrated with other control tactics on an area-wide basis. The target insect is reared in large numbers in specialised factories, the male sex sterilized with ionizing radiation and sequentially released in the target area in numbers that allow them to outcompete their wild counterparts for mates. Mating of a sterile male with a virgin wild female results in no offspring and the reduced replacement rate will eventually suppress or even eradicate a target population [19,20].

Spiking bloodmeals with an insecticide for the purpose of mosquito sex separation is not a new idea. This method was implemented in an *An. albimanus* control program with an SIT component in El Salvador, whereby >95% of females were eliminated by adding malathion to bloodmeals [21]. Although not all females could be eliminated and increased male mortality was a negative side effect, this method was applied for lack of a better alternative until it was replaced by a genetic sexing strain (GSS) based on propoxur resistance [21] in the males.

A number of other substances have also shown to reduce mosquito longevity; however, for the purpose of female elimination in a mass-rearing setting for the SIT, the fatal effects of these compounds need to be quick and reliable, resulting in 100% female kill, while having no detrimental effects on male mating efficiency following their release. We therefore tested substances in addition to ivermectin and malathion that may be suitable, and have been previously reported as having lethal effects in mosquitoes or other insects: spinosad, a known insecticide with acute oral toxicity in honeybees [22] and has been tested for attractive toxic sugar baits (ATSB) for mosquito control [23]; boric acid which was also used effectively for ATSB against *An. gambiae* and *An. arabiensis* in Mali [24]; dieldrin which has been used in previous studies by Yamada *et al.*[25] and has shown to have high contact toxicity in *An. arabiensis*, and finally household detergent to assess if it had any lethal effects when ingested by adult mosquitoes (as opposed to its effects when applied to larval stages, [26]).

The following experiments were designed to test the various toxicants in bloodmeals to determine whether they can eliminate *An. arabiensis* females quickly and reliably. In addition, further investigations were conducted to determine the mating efficiency of the remaining males in terms of insemination rates of new, virgin females made available to these males. Based on these results the most efficient toxicant was then used on a larger scale (n = 1000 insects) including irradiation of the insects as pupae to assess the feasibility of this female elimination method in the context of the SIT.

## Methods

*Mosquito colony rearing: An. arabiensis* originating from Dongola, Northern State, Sudan, were reared in the insectary as described by Yamada *et al.*[25].

### Preliminary tests: selecting a toxicant

Blood meals (defrosted bovine blood) were prepared in 45 ml centrifuge tubes containing the following substances at various concentrations: Detergent Pril Original (Henkel KGaA, Düsseldorf, Germany) (1000 ppm, 5000 ppm, 10000 ppm (0.1, 0.5, and 1% solutions)), boric acid (Sigma Aldrich Handels GmbH, Vienna, Austria) (1000 ppm, 5000 ppm, 10000 ppm), dieldrin (Sigma Aldrich Handels GmbH, Vienna, Austria) (1, 1.5 and 2 ppm solutions), malathion (Sigma Aldrich Handels GmbH, Vienna, Austria) (0.5, and 2 ppm solution), ivermectin (Virbamec, Virbac Oesterreich GmbH, Vienna, Austria) (0.5, 2, 5, and 7.5 ppm solutions), and spinosad (spynosin A and D, Sigma Aldrich Handels GmbH, Vienna, Austria) (1, 5, and 10 ppm solutions). The blood was warmed in a water bath and subsequently poured into a Hemotek device that maintained the temperature of the blood at 35°C ± 1°C and placed on 30 × 30 × 30 cm cages containing 100 mosquitoes for a period of 2 h. Dead mosquitoes were counted each morning over the following 5 days. The toxicants most effective in eliminating females were selected for use in a subsequent larger-scale trial.

### Optimising the efficiency of eliminating females and assessing the effect on male mating

Nine standard 30 × 30 × 30 cm cages (Megaview Science Education Services Co, Ltd, Taiwan) were stocked each with 1000 pupae (a mix of males and females): three for treatment with 7.5 ppm ivermectin-spiked blood, three for treatment with 10 ppm spinosad-spiked blood, and three control cages receiving untreated, defrosted bovine blood. Blood meals prepared as described above were offered to the mosquitoes for at least 2 h every day. Mortality of male and female mosquitoes was assessed every morning until all females had died.

After all females were eliminated from the treatment groups (day 4 post emergence), 100 males from each ivermectin cage (as this worked more efficiently than spinosad) and from each control cage were transferred to new cages, each containing 100 virgin females of the same age as the males. Some of these males from the ivermectin treatment cages were likely to have mated with a small proportion of the few remaining females before they were all eliminated, whereas the males from the control cages were likely to have mated for the entire duration (4 days) of the experiment as these females were not being killed. Additional three cages were stocked with 100 virgin males and 100 virgin females for evaluation of the mating potential compared to the males from the treatment cages. All adult mosquitoes were of the same age. They were left to mate for three consecutive nights, before the females were removed and dissected under a stereoscope and checked for insemination: females were collected and spermathecae were checked for the presence of sperm. Insemination rates were calculated as the number of females inseminated over total number of females.

### Testing the feasibility of blood spiking in the context of the SIT

*An. arabiensis* mosquitoes were reared as described above, and batches of approximately 1000 pupae that had been quantified volumetrically in netted spoons were irradiated (20–26 h after pupation) in a ^60^Co Gamma cell 220 (Nordion, Ottawa, Canada) with a dose of 70 Gy which induces partial sterility (~83%) as described by Helinski *et al.*[27]. A dosimetry system was used to verify the dose received by the batches based on the Gafchromic HD-810 film (International Speciality Products, NJ, USA) [28]. Each group of pupae was then placed in each of 9 (30 × 30 × 30 cm) cages (3 for ivermectin treatment, 3 control cages, and 3 cages with virgin sterile males only) and allowed to emerge. Pupal mortality was recorded. An additional batch of 1000 non-irradiated pupae was sexed and both males and females were kept for the mating efficiency experiments.

Adult mosquitoes were offered a blood meal daily with untreated blood (control), or with 7.5 ppm ivermectin spiked blood as described above, and mortality counted daily until all females were dead. Cages with only virgin males were not offered a bloodmeal.

After all females were eliminated from the treatment cages, 100 males from each of the 3 treatment cages, each of the 3 control cages, from the 3 cages of virgin sterile males, and from the 3 cages of virgin fertile males were aspirated out and transferred to 12 new separate cages all containing 100 virgin females. All adults were of the same age. They were left to mate for 3 nights before the females were removed and dissected to check for insemination as described above. The cages containing virgin males only were given a sugar solution only.

The 4 types of males were compared in terms of mating efficiency: The sterile males from the control cages that were kept together with females for the 4 blood feeding days (C ♂); the sterile males from the treatment cages remaining after all females were eliminated by spiked bloodmeals (Tx ♂); virgin sterile males that were kept separately from females and had no chances of mating (v.♂ ster); and virgin fertile males that were kept separately from females and had no chances of mating (v.♂ fert).

### Statistical analysis

The reduction of female longevity in all treatment groups was compared using Kaplan-Meier survival analysis. The resulting survival curves were then compared to the control using Mantel-Cox log-rank tests. Insemination rates for each of the test groups were pooled to get an average value per treatment, and were compared by ANOVA. Graphics and statistical analyses were performed using Microsoft Excel 2003 (Microsoft, 216 Redmond, WA; 1985–2003) and Minitab release 13.32 (Minitab; 2000). In all cases, the alpha level was *P* < 0.05.

## Results

### Selecting toxicant

In the samples of 100 adults, ivermectin and spinosad eliminated females most quickly and thoroughly, with 100% kill by day 4 (Table 1). Spinosad at 10 ppm was able to knock down females within 12 h of the bloodmeal, but they took longer to die than the ivermectin bloodmeals, which knocked down females almost immediately after blood-feeding, and killed them within 12 h, resulting in almost 80% female kill after the first bloodmeal. Based on these results spinosad (10 ppm) and ivermectin (7.5) were selected as the most promising and used for the following experiment.

**Table 1:** Population reduction of males and females following bloodmeals containing 6 different toxicants compared to controls fed on untreated bovine blood

**Toxicant**	**Concentration**	**Day**	**1**	**2**	**3**	**4**	**5**
		**Sex**	**F / M**	**F / M**	**F / M**	**F / M**	**F / M**
Control	0.00%	F	0.00	0.02	0.02	0.04	0.04
		M	0.00	0.00	0.02	0.02	0.06
	0.00%	F	0.02	0.06	0.06	0.08	0.08
		M	0.00	0.02	0.04	0.04	0.06
	0.00%	F	0.00	0.03	0.03	0.05	0.13
		M	0.00	0.00	0.00	0.00	0.03
Detergent	0.10%	F	0.02	0.02	0.02	0.02	0.06
		M	0.00	0.00	0.00	0.02	0.06
	0.50%	F	0.00	0.00	0.00	0.06	0.08
		M	0.02	0.02	0.02	0.06	0.08
	1.00%	F	0.02	0.02	0.02	0.06	0.14
		M	0.00	0.00	0.00	0.02	0.04
Boric acid	0.10%	F	0.00	0.00	0.00	0.12	0.22
		M	0.00	0.00	0.06	0.10	0.14
	0.50%	F	0.00	0.04	0.08	0.24	0.32
		M	0.00	0.00	0.02	0.02	0.06
	1.00%	F	0.00	0.02	0.32	0.50	0.66
		M	0.00	0.00	0.04	0.06	0.08
Dieldrin	1 ppm	F	0.00	0.66	0.66	0.79	0.81
		M	0.06	0.07	0.07	0.07	0.09
	1.5 ppm	F	0.00	0.72	0.72	0.76	0.83
		M	0.06	0.06	0.06	0.06	0.07
	2 ppm	F	0.00	0.79	0.79	0.83	0.84
		M	0.06	0.08	0.09	0.13	0.14
Spinosad	1 ppm	F	0.00	0.04	0.20	0.42	0.42
		M	0.00	0.02	0.06	0.06	0.06
	5 ppm	F	0.12	0.26	0.34	0.84	0.84
		M	0.00	0.02	0.06	0.06	0.06
	**10 ppm**	**F**	**0.32**	**0.42**	**0.76**	**1.00**	**1.00**
		**M**	**0.00**	**0.00**	**0.04**	**0.04**	**0.06**
Ivermectin	0.5 ppm	F	0.18	0.20	0.25	0.48	0.48
		M	0.00	0.00	0.03	0.08	0.08
	2 ppm	F	0.35	0.45	0.53	0.78	0.83
		M	0.00	0.00	0.00	0.03	0.05
	**5 ppm**	**F**	**0.79**	**0.97**	**0.99**	**1.00**	**1.00**
		**M**	**0.00**	**0.00**	**0.02**	**0.02**	**0.04**
Malathion	0.5 ppm	F	0.00	0.08	0.10	0.15	0.18
		M	0.00	0.00	0.00	0.00	0.05
	2 ppm	F	0.00	0.13	0.18	0.35	0.58
		M	0.00	0.03	0.03	0.08	0.08

Substances most effective in killing females, while not affecting males are shown (in **bold**). Each sample consisted of 100 adult mosquitoes (50 females and 50 males).

### Optimising the efficiency of eliminating females and assessing the effect on male mating

#### Spiking bloodmeals

**Figure 1:**
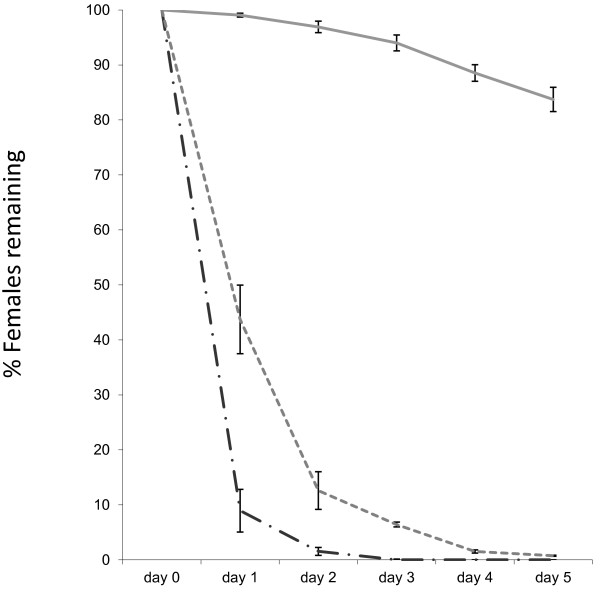
**Percentage of females remaining in control cages and treatment cages.** Elimination of females from the cage population (approximately 500 females and 500 males) over 5 days in control cages (solid line), in cages fed with 10 ppm spinosad-spiked blood (dotted line), and 7.5 ppm ivermectin-spiked blood (semi-dotted line). Error bars represent SEM.

**Figure 2:**
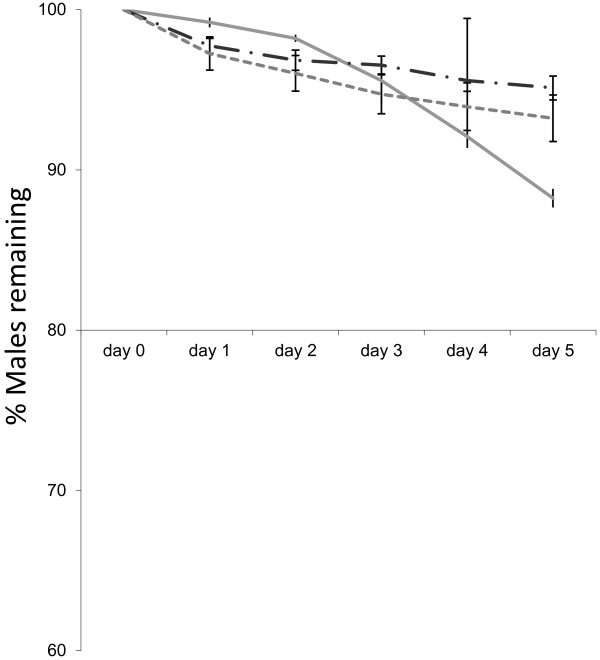
**Percentage of males remaining in control cages and treatment cages.** Males remaining in cages for the duration of 5 days in control cages (solid line), in cages fed with 10 ppm spinosad-spiked blood (dotted line), and 7.5 ppm ivermectin-spiked blood (semi-dotted line). (Cages contained 500 females and 500 males). Error bars represent SEM.

The adult female mosquitoes readily took a bloodmeal on day 1 post emergence. Of the samples of 1000 adults (volumetrically estimated as pupae and with a male:female ratio close to 1:1), approximately 10% of the females did not take a blood meal on the first day but both ivermectin and spinosad worked well in eliminating females from the cages. However, comparing daily mortalities, ivermectin at 7.5 ppm was faster in knocking down and killing the females than the spinosad at 10 ppm (day 1: *p =* 0.0091; day 2*: p =* 0.0342; day 3*: p =* 0.0001; day 4: *p =* 0.0057). Ivermectin was able to kill more than 90% of females on day 1, more than 98% by day 2, and 100% by day 3, while spinosad needed approximately one day longer to achieve similar results (Figure 1). The results indicate that approximately 16% mortality occurred amongst control females, reflecting a normal level of mortality as observed in regular rearing after blood feeding. Males in both treatment groups survived well during the 5 day experimental period, with only 5% mortality in the ivermectin cages and 7% in the spinosad cages (Figure 2). Control cages had higher male mortality (12%, *p =* 0.0071).

#### Mating efficiency

**Figure 3:**
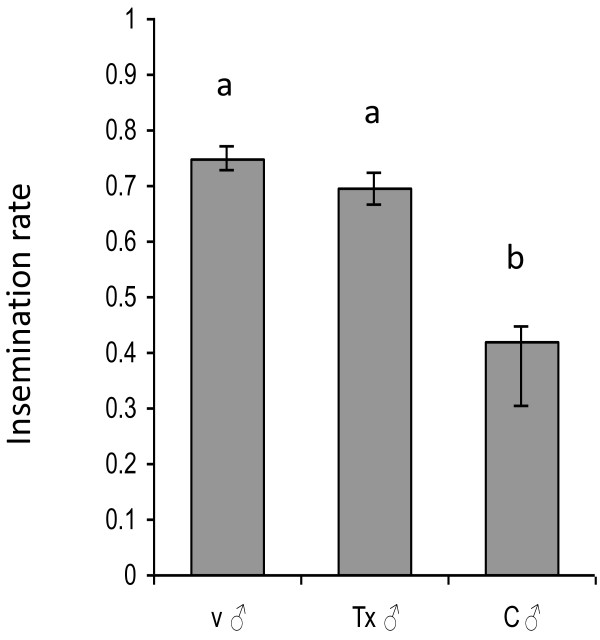
**Insemination rates of females by virgin males (v ♂), males from treatment cages (ivermectin) (Tx ♂), and males from control cages (C ♂).** Each group consisted of 100 males and 100 females. Groups that are statistically different are labelled with different letters. Error bars represent SEM.

Males from the female treatment group were as efficient (69.5%) *(p =* 0.146*)* in inseminating virgin females as were virgin control males (74.8%) (Figure 3). Males in the control cages were significantly less efficient in inseminating the new virgin females added to them (41.7%), than were males from the treatments and virgin males *(p =* 0.016*, p =* 0.007*).*

### Testing the feasibility of blood spiking in the context of the SIT

#### Irradiation at the pupal stage

Among irradiated and non-irradiated pupae mortality rates were slightly elevated (close to 2%) possibly due to the volumetric quantification process. Close to 1000 adults emerged in all cages at an approximate 1:1 male to female ratio as would be expected in nature.

#### Spiking bloodmeals

**Figure 4:**
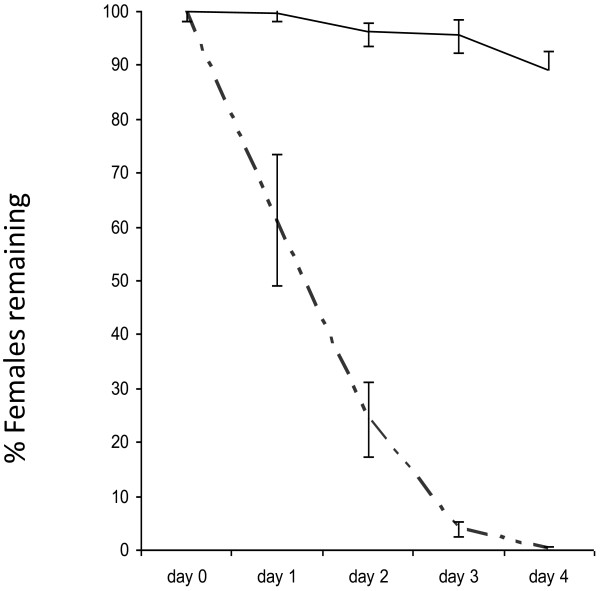
**Percentage of irradiated females remaining in control cages and treatment cages.** Elimination of irradiated females from the population (approximately 500 females and 500 males) over 4 days in control cages (solid line) and in cages fed with 7.5 ppm ivermectin-spiked blood (semi-dotted line). Error bars represent SEM.

**Figure 5:**
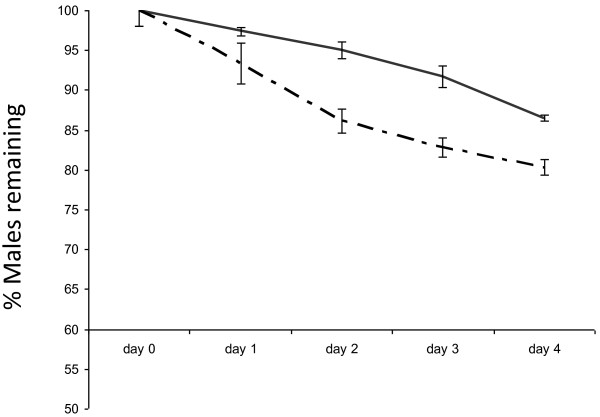
**Percentage of irradiated males remaining in control cages and treatment cages.** Irradiated males remaining in cages for the duration of 4 days in control cages (solid line) and in cages fed with 7.5 ppm ivermectin-spiked blood (semi-dotted line). Error ba represent SEM.

In this trial, it was still possible to eliminate almost all females (96%) by day 3, and 100% by day 4 (Figure 4), however, the number of irradiated females killed during the first 2 days was much lower than in the trial with non-irradiated females (38.8% on day 1, and 75.7% on day 2, compared to almost 90% on day 1, and 98% on day 2, respectively). Male mortality in the treatment cages was slightly, but significantly higher at 20% (*p =* 0.0041) than in the control cages (14%) (Figure 5).

#### Mating efficiency

**Figure 6:**
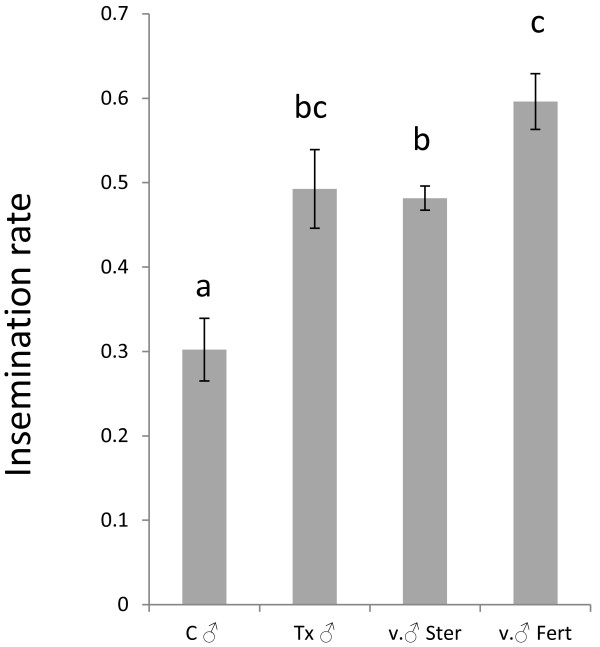
**Insemination rates of females by irradiated males from control cages (C ♂), irradiated males from ivermectin treatment cages (Tx ♂), irradiated virgin males (v. ♂ Ster), and fertile virgin males (v. ♂ Fert).** Each group consisted of 100 males and 100 females. Groups that are statistically different are labelled with different letters. Error bars represent SEM.

The results show that C ♂ were unable to mate with as many females as observed in the other groups, with only 30% of the females inseminated (Figure 6). The “optimal” males, v.♂ fert, were only able to inseminate an average of 59.6% of the females in the 3 night period. Both the Tx ♂ and v.♂ ster were able to mate with, and inseminate approximately half of the females (49.2% and 48.1% respectively).

## Discussion

The *An. arabiensis* females readily took a blood meal from day 1, which is not always observed in other mosquito species. Those that fed on the ivermectin-spiked blood were killed reliably and efficiently within 12 hours. No instance of females surviving a sub-lethal dose was observed. At these concentrations, even non-fully engorged females were killed. The challenge is to motivate as many of the females as possible as soon as possible to take a blood meal. Therefore, improving the blood-feeding method may directly increase female killing, for instance removing the sugar source several hours before offering the blood-meal, using fresh blood as opposed to thawed frozen blood, timing the blood-feeding to preferred times of day (for example dusk/dawn, or during the night), prolonging blood-feeding for several hours, or feeding multiple times per day, keeping in mind not all of these suggestions are practical or cost effective in a mass-rearing setting. Some of the treatment cages contained adult females that did not take a blood meal at all (approximately 1 in 500, i.e. 0.002%). These were few, and mostly unusually small in size, and as they refuse to blood feed, (even when offered arm-feeding by a volunteer), they would not pose a threat as a disease transmitter. However, it has been reported for other species, that virgin females sometimes do not blood feed initially, but then may later take a blood meal once inseminated. It was found by Charlwood *et al.*[29] that in the wild, the size of female *An. gambiae* is important but not completely responsible for them feeding as virgins. Most of the smaller females survived to mate, but did not seem to survive for very long thereafter [29]. Whether *An. arabiensis* females that refuse bloodmeals initially go on to mate and subsequently seek a blood meal is not known, and would require further research. Furthermore, if females were to survive bloodmeals that perhaps have a sublethal concentration of ivermectin (due to possible miscalculations while preparing the blood meals), it has been reported that ivermectin in the mosquito disrupts parasite development, thereby reducing the female’s disease transmitting potential [5].

For sterile male releases in the context of the SIT, the complete elimination of females from a mosquito population at the adult stage as is the case here needs to be quick. The longer the females are present in the cages, the higher the chances of their mating sterile males, which are unable to replenish their sperm stock, making them less efficient in inseminating wild females post-release and therefore compromising the SIT’s effectiveness. However, there have been some studies suggesting that irradiated mosquitoes require some days to recover before regaining mating activity [30], making the few days needed for 100% female kill not necessarily detrimental to male mating efficiency post release. The implications of keeping the adult males for several days before releasing them have not yet been fully investigated. Allowing adult males to sugar feed, mature sexually, and recover from handling and irradiation before mass releases may indeed improve their competitiveness compared to releasing them as pupae, or immediately after emergence.

The observation of some mortality in both males and females in control cages were not unexpected as overcrowding cages and regular blood-feeding has been shown to cause elevated mortality rates in insectary rearing. Male mortality was low in treatment cages, but survival may still be improved by adding resting sites and additional sugar sources.

Male survival is equally a priority for the SIT. Therefore, the toxicants to be chosen for female killing should ideally induce high oral- and low contact toxicity. In a mass-rearing facility, large amounts of the toxicant may be required, therefore the substance should be relatively inexpensive, readily available, and most importantly, safe for humans, other vertebrates and the environment. Some of the substances tested in this experiment did not meet these requirements, were less effective than ivermectin and should therefore not be considered for mosquito sexing purposes on a large scale. Both ivermectin and spinosad are good candidates as they can be considered a natural product that are used safely in organic agriculture [31]. They have a good environmental profile, and both substances have low vertebrate toxicity, making them relatively safe for human handling.

The mating frequency of the first batch of non-irradiated males from the treatment environment was acceptable considering that there was a 1:1 male:female ratio on the first day post-emergence. This can be explained by the males not having reached full sexual maturation at this point, resulting in minimal to no mating activities during the first day. On the second day, the male:female ratio was closer to 10:1 with only few females available for mating. By day 3, there were no more females available. When these males were added to virgin females again at a 1:1 ratio, they were able to mate as efficiently as the virgin control males in the 3 day mating period. However, it is important to note that fertile *An. arabiensis* males are able to replenish their sperm stock for a period of at least 6 days [32]. Nevertheless, fertile males from control cages were far less efficient in mating the virgin females after spending 4 days together with females during the experiment although they had a 3 day mating period in which sperm production and mating could take place.

Post irradiation, there was an indication that the females did not take a blood meal as readily on the first 2 days as the non-irradiated females in the previous experiment. This could be due to the need for a recovery phase after the irradiation process [30]. The irradiation did not, however, increase mortality in control females during the first 4 days post-emergence. Although by day 3, most of the females (96.1%) in the treatment groups had been killed, there was less blood-feeding and thus less mortality among females during days 1 and 2. This allowed females to remain available for mating, which in theory may decrease male mating potential after this period considering that fully sterile males are no longer able to produce new sperm and can mate only a few times [32]. Partially sterile males may show similar characteristics in terms of mating abilities and limited sperm production, but this requires further investigation.

As initially expected, the mating efficiency of the partially sterile males from the control cages achieved lower insemination rates in the new virgin females as they had the opportunity to mate with the surviving females for the duration of the 4 blood feeding days. Nevertheless, the males were able to mate with 30% of the virgin females, suggesting that the peak mating activity may be delayed by the effects of irradiation, or partially sterile males are able to mate more often than initially expected. More surprisingly, treatment of males and virgin sterile males showed no difference in their mating capabilities in terms of the proportion of new virgin females inseminated. Although there were still females present in the treatment cages on days 1 and 2, a delay in mating activity until after these days could again explain that there was little to no mating in the treatment cages. Control virgin fertile males were able to inseminate around 60% of the virgin females in the 3 nights. The 10% decrease in the insemination rates seen in both the treatment and virgin sterile males may be attributed to their partial sterility only, with little or no impact on mating potential from the female elimination process.

## Conclusions

Ivermectin is an acceptable substance in terms of environmental and health safety, and is very efficient in killing female *An. arabiensis* following oral exposure via spiked bloodmeals. It is possible to eliminate all females quickly, allowing the remaining males to sustain their mating potential following release, even after irradiation at 70 Gy. Further tests are needed to ensure these capabilities following a fully sterilising dose of 120 Gy [27,33]. Alternatively, the approach we describe here followed by male irradiation at the adult stage is an option that may improve both blood-feeding behaviour in females, and male fitness post release, as it has been reported that irradiation at later (adult) stages incurs less somatic damage thus improving competitiveness in mosquitoes [27]. This method requires further evaluations on the mating potential and competitiveness of males remaining after blood spiking that will be irradiated versus wild males. All in all, the method of female elimination by spiking blood meals discussed here has room for improvement, but has the potential for use in SIT, IIT, or other techniques requiring simple and effective female elimination.

Although the method described here will likely be practical as a temporary solution and for lack of better alternatives, it does, however, have limitations. Keeping females until a blood feeding age in a mass-rearing facility will increase the costs significantly compared to rearing only males until adulthood in terms of feeding, as well as space and labour requirements. In addition, the reliability of the method will not remain at a constant 100% as it is dependent on behavioural attributes, which can never be controlled completely, and may change over time or in response to other external factors. The efficiency and reliability of a good genetic sexing strain will be difficult to obtain, but this method is effective, simple, and has potential for application against many other mosquito species that currently lack alternative sexing methods.

## Competing interests

The authors declare that they have no competing interests.

## Authors’ contributions

HY conceived of and designed the study, carried out the experiments and drafted the manuscript. SMS assisted in the development of the experiment protocols, the rearing and supply of the mosquitoes, and the dissection of the female mosquitoes. MJBV and DDC contributed substantially in the development of the manuscript. JLRG oversaw the project as group leader. All authors read and approved the final version of the manuscript.
